# Effects of Temperature, Axial Ligand, and Photoexcitation on the Structure and Spin-State of Nickel(II) Complexes with Water-Soluble 5,10,15,20-Tetrakis(1-methylpyridinium-4-yl)porphyrin

**DOI:** 10.3390/molecules29020310

**Published:** 2024-01-08

**Authors:** Máté Miklós Major, Zsolt Valicsek, Ottó Horváth

**Affiliations:** Research Group of Environmental and Inorganic Photochemistry, Center for Natural Sciences, Faculty of Engineering, University of Pannonia, P.O. Box 1158, H-8210 Veszprém, Hungary; miklos.mate.major@gmail.com (M.M.M.); valicsek.zsolt@mk.uni-pannon.hu (Z.V.)

**Keywords:** cationic nickel(II) porphyrin, square planar, high spin, low spin, spin-state equilibrium, photoinduced shift, photophysics, associate with TEOA

## Abstract

Water-soluble metalloporphyrins, depending on the metal center, possess special spectral, coordination, and photochemical features. In nickel(II) porphyrins, the Ni(II) center can occur with low-spin or high-spin electronic configuration. In aqueous solution, the cationic nickel(II) complex (Ni(II)TMPyP^4+^, where H_2_TMPyP^4+^ = 5,10,15,20-tetrakis(1-methylpyridinium-4-yl)porphyrin), exists in both forms in equilibrium. In this study, an equilibrium system involving the low-spin and high-spin forms of Ni(II)TMPyP^4+^ was investigated via application of irradiation, temperature change, and various potential axial ligands. Soret band excitation of this aqueous system, in the absence of additional axial ligands, resulted in a shift in the equilibrium toward the low-spin species due to the removal of axial solvent ligands. The kinetics and the thermodynamics of the processes were also studied via determination of the rate and equilibrium constants, as well as the ΔS, ΔH, and ΔG values. Temperature increase had a similar effect. The equilibrium of the spin isomers was also shifted by decreasing the solvent polarity (using n-propanol) as well as by the addition of a stronger coordinating axial ligand (such as ammonia). Since triethanolamine is an efficient electron donor in Ni(II)TMPyP^4+^-based photocatalytic systems, its interaction with this metalloporphyin was also studied. The results promote the development of efficient photocatalytic systems based on this complex.

## 1. Introduction

**Figure 1:**
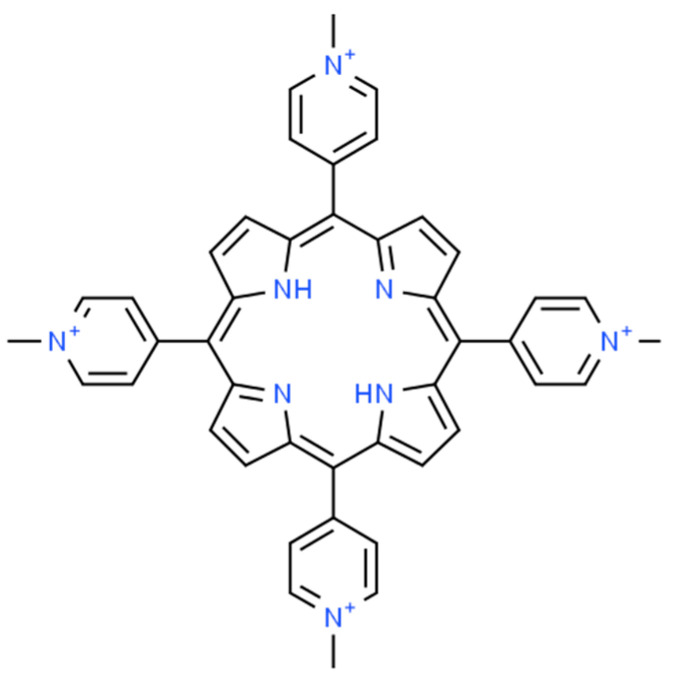
The structure of the free-base ligand (H_2_TMPyP^4+^ = 5,10,15,20-tetrakis(1-methyl-4-pyridinium-)porphyrin).

Water-soluble metalloporphyrins, due their special spectral, coordination, and photoredox features, offer numerous possibilities of application in various photochemical procedures, e.g., for visible light-driven photocatalysis [1,2,3,4,5,6]. These peculiar coordination and electronic properties are due in large part to the structure of the porphyrin ligand. The basic compound is porphin, a ring of four pyrroles linked through four methine groups. In the center of the resulting macrocycle are four nitrogen atoms (two acidic and two basic) in a nearly planar arrangement, which allows the molecule to bind metal ions (Figure 1). The position (spacing) of the pyrrolic nitrogen atoms is approximately constant due to the rigidity of the aromatic macrocycle and therefore limits the coordination of the metal ion. On this basis, two broad groups are distinguished:

- Planar or normal metal porphyrins are formed when the radius of the coordinating metal ion is less than about 80–90 pm.
- Out-of-plane porphyrins (OOP = out of plane or SAT = sitting atop) are formed when the radius of the coordinating metal ion is larger than 80–90 pm, so it is displaced from the coordination cavity, distorting it [7].

The two groups significantly differ in their photophysical, electronic, and coordination properties. Among the out-of-plane (e.g., lanthanide) complexes, the ligand undergoes photo-oxidation and thus (transient) reduction of the central metal ion upon light absorption [8]. A large group of important reactions is those of photo-redox systems, in which various, usually normal metalloporphyrins often act only as sensitizers, e.g., in photodynamic therapy [9,10,11]. In addition to photosensitization, research is ongoing to design homogeneous and heterogeneous photocatalytic systems. The mechanism of the photoredox catalytic reactions may also differ depending on whether the whole macrocycle or only the central metal ion is involved in electron transfer. Our research group has successfully created functional photo-reductive systems using cationic manganese(III) and cobalt(III) porphyrins [12]. The photoreduction of the complexes was achieved by electron uptake of the central metal ion.

The porphyrin complexes of nickel can be found in both natural and artificial forms. Abelsonite belongs to the previous group. It is an anisotropic nickel porphyrin mineral and is associated with the degradation of chlorophyll [13]. It is also part of the naturally occurring nickel(I)-tetrapyrrole cofactor F430, which functions by promoting the formation of the nickel(I) (d^9^) form and the nickel(III) (d^7^) transition form by a partially saturated porphyrin ligand [14].

A number of artificial nickel porphyrins have been investigated [15,16] due to the catalytic properties of the aforementioned cofactor F430. One of them is Ni(II)OEP (OEP = octaethylporphyrin), which was studied by Dongho Kim et al. [17]. In their research, the addition of piperidine to Ni(II)OEP dissolved in trichloromethane led to a significant change in its absorption pattern, which they explained was due to spin isomerization and the introduction of two axial ligands. However, the absorption spectra recorded during the process showed a linear increase in the concentration of the emerging complex with the ligand, which would have led to the conclusion that only one axial ligand was present instead of two. In the absence of axial ligands, nickel(II) porphyrins possess a diamagnetic low-spin state due to the doubly occupied d_z_2 orbital of the metal center. If at least one axial ligand is coordinated, one electron is transferred from this orbital to the d_x2-y2_ one, resulting in a paramagnetic high-spin complex [18]. Notably, the ligand exchange is very fast, and, generally, the first association constant (regarding the formation of the five-coordinate complex) is lower than the second one [17,19,20].

The structure of nickel(II) tetraphenylporphyrin was investigated by both quantum chemical and analytical methods [21]. Different results were obtained by Raman measurements excited at various wavelengths, from which the presence of several forms of porphyrin in solution was inferred. In a two-particle conjecture, one is a planar structure, the other a distorted structure, with the latter either only “ruffled” or a complex with both “ruffled” and “saddled” distortions.

Ni(II)TMPyP^4+^ (H_2_TMPyP^4+^ = 5,10,15,20-tetrakis(1-methylpyridinium-4-yl)porphyrin, Figure 1), studied in this work, was previously investigated by Pasternack and Spiro [22]. They observed the presence of a complex, double Soret band in aqueous media. No evidence of dimer formation was observed with increasing concentration or temperature. Their NMR studies showed that one of the two forms of porphyrin was a high-spin one with a coordination number of five or six, whereas the other was a low-spin form with a coordination number of four. There was no experimental evidence for the former, octahedral structure, only its higher abundance, but it was noted that, for example, Ni(II)TPP was present under certain conditions with a coordination number of 5. The authors justified the coordination number of 6 with the two isosbestic points appearing in the absorption spectra in the presence of a strong nitrogen base (imidazole). According to them, the second isobestic point (at a rather high (1.5 M) concentration of imidazole) corresponded to the entry of the second axial ligand, but this was only indicated by the intersection of three spectra, and the high imidazole concentration may have caused a solvent effect, decreasing the reliability of the conclusion. The equilibrium system was also investigated in the presence of acetone, and it was found that the equilibrium was shifted towards the formation of the low-spin complex, but several contradictions were found in this study [22].

Following basic equilibrium studies of Ni(II)TMPyP^4+^, the two forms of its appearance were also studied by Raman spectroscopy, and differences in the range of skeletal vibrations were observed, but the number of the axially coordinating ligands were not revealed [23]. The kinetically inert Ni(II)TMPyP^4+^ complex, similarly to the corresponding manganese(III) and cobalt(II) hyper porphyrins, displayed peculiar photophysical as well as advantageous electron relay properties [12].

In the presence of a suitable electron donor (triethanolamine, TEOA) and acceptor (methylviologen, MV^2+^), these metalloporphyrins proved to be efficient photocatalysts, transferring electrons between the ground-state donor and acceptor via the outer-sphere mechanism. In these systems, triplet excited-state Mn(III) and Co(III) porphyrins are dynamically quenched with TEOA. In the case of the nickel(II) complex, however, this electron donor forms an associate with Ni(II)TMPyP^4+^ in a ground-state equilibrium. The lifetime of the triplet excited state of this species is much longer than that of nickel(II) porphyrin alone [24]. This deviating behavior is partly related to the spin state of the metal center being very sensitive to the potential axial ligands in the solution. The aim of our work was to demonstrate how the spin state, the structure, and the photophysical properties of this complex can be influenced by the potential axial ligand and how the temperature and photo-excitation can affect the equilibrium of these states.

## 2. Results and Discussion

### 2.1. Photophysical Properties and Spin States

**Figure 2:**
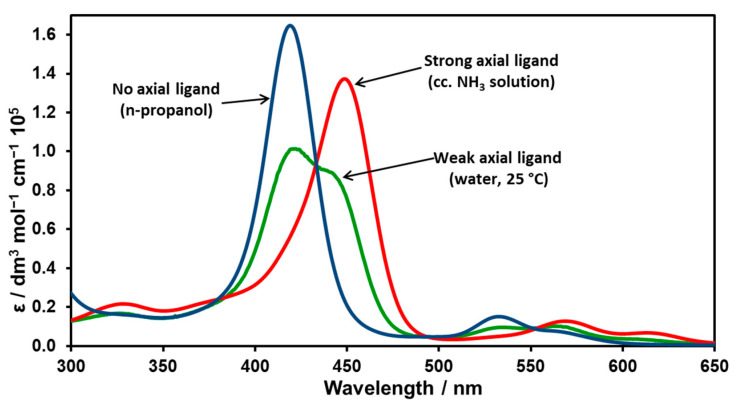
Soret bands of Ni(II)TMPyP^4+^ in various media.

Depending on the medium and the presence of potential axial ligands, nickel(II) porphyrins can occur with a low-spin metal center (in apolar, non-coordinating solvents) or with a high-spin metal center (in the presence of strongly coordinating axial ligands). In water, both species exist in equilibrium, per Equation (1) (Figure 2).

Ni(II)TMPyP^4+^ + H_2_O ⇔ [Ni(II)TMPyP(H_2_O)]^4+^ (1)

Since water is a weakly coordinating axial ligand compared to NH_3_ and, especially, piperidine, only one H_2_O molecule can be coordinated in this case. Thus, to determine the spectra for different spin states of Ni(II)TMPyP^4+^, this equilibrium must be shifted so that only one or the other complex is present as clearly as possible. To be able to investigate complexes with and without axial ligands separately, the polarity of the medium was changed. First, n-propanol was added to the stock solution of the nickel complex; it reduced the dielectric constant of the medium and created an apolar microphase around the lipophilic interior of the porphyrin. N-propanol is a weaker-field ligand than water, so it was possible to shift the equilibrium and inhibit axial coordination. A Soret band at 420 nm was obtained in the absorption spectrum (Figure 2). The resolution of the spectrum is provided in Table 1.

Another sample of Ni(II)TMPyP^4+^ was treated with a concentrated ammonia solution. This caused an intense Soret band at 447 nm to appear in the absorption spectra. This phenomenon suggests that NH_3_ was axially coordinated, creating a high-spin complex. The resolution of the spectrum is given in Table 2.

The redshift of the Soret band in the case of the high-spin complex can be attributed to the increase in the size of the metal center. Thus, in the case of a five-coordinate complex (with square pyramidal geometry), the size of Ni^2+^ would exceed that of the cavity of the macrocycle, and therefore, the metal center would be located slightly outside of the ligand plane, distorting it. Notably, in the case of a very strongly coordinating ligand, in the presence of which a six-coordinate octahedral complex is formed, the distortion is an expansion of the porphyrin ring caused by the increased size of the metal center.

**Figure 3:**
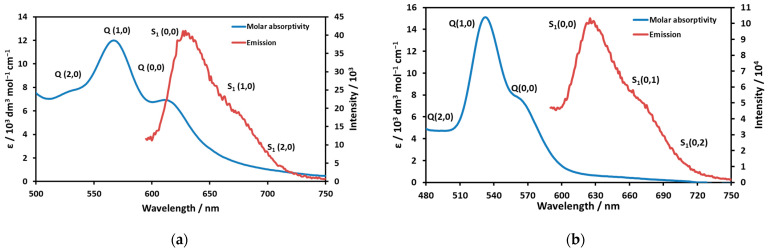
(**a**) Molar absorptivity of Q-bands and S1 fluorescence of the high-spin Ni(II)TMPyP^4+^ complex. (**b**) Molar absorptivity of Q-bands and S_1_ fluorescence of the low-spin Ni(II)TMPyP^4+^ complex.

Ni(II)TMPyP^4+^ displayed a characteristic S_1_ fluorescence. Interestingly, the emission spectra were very similar for the two species with different spin states, whereas the absorption (Q-) bands deviated considerably (Figure 3a,b).

**Figure 4:**
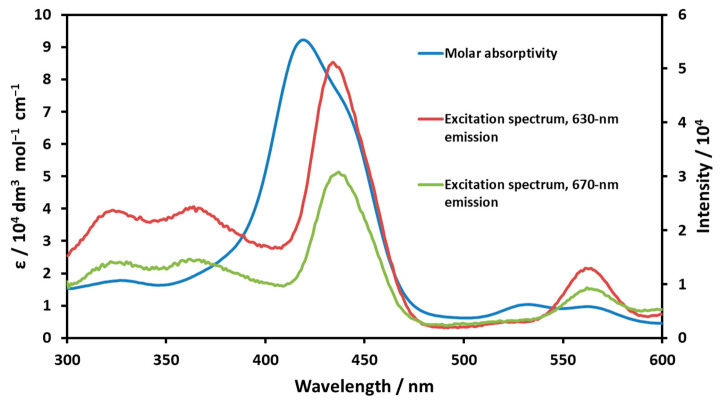
Excitation and absorption spectra of Ni(II)TMPyP^4+^ in water, an equilibrium system involving both high-spin and low-spin species.

The emission spectrum of the high-spin complex (gained by excitation at various wavelengths) was resolved into three bands: S_1_(0,0) 630 nm, S1(0,1) 675 nm, and S_1_(0,2) 722 nm. The Stokes shift, i.e., the energy difference between the Q(0,0) absorption and the S1(0,0) emission bands, was 396 cm^−1^, which suggests a moderate structural change during the excitation. The band maxima of the resolved emission spectrum of the low-spin complex were S_1_(0,0) 628 nm, S_1_(0,1) 668 nm, and S_1_(0,2) 700 nm. In this case, the formal Stokes shift would be 1830 cm^−1^, which is unrealistically large. This value, along with the very similar emission spectra and the previously published emission lifetimes (1.36 ns and 1.19 ns for the low-spin and high-spin species, respectively [24]), indicate that both emissions originated from the same species. Since the Stokes shift for the low-spin species (on the basis of the corresponding absorption and emission band maxima) would be unrealistically large and its fluorescence intensity was significantly lower than that for the high-spin species, in the case of this complex, the emission of the high-spin one, which existed in a small fraction, was detected. In order to confirm this conclusion, the excitation spectra were also recorded and compared to the corresponding absorption spectra. In the case of the high-spin complex, the absorption and excitation spectra were very similar (Figure S1 in the Supplementary Materials), whereas they considerably differed for the low-spin species (Figure S2). Notably, in the excitation spectrum of the low-spin complex (the sample with high n-propanol concentration), a band appeared at about 615 nm, which also indicated the presence of the high-spin species. The excitation spectrum of Ni(II)TMPyP^4+^ (compared to the absorption one) in water provided additional proof of our conclusion (Figure 4).

In the range of the Soret bands, excitation spectra (at 630 and 670 emission wavelengths) overlapped well with the absorption band of the high-spin species in the equilibrium, and the position of this excitation band unambiguously deviated from that of the Soret band of the low-spin complex. These observations indicate that the low-spin complex, the geometry of which was square planar, did not perceptively fluoresce. Since this kind of emission originated from the porphyrin ligand, the low-spin Ni(II) center very efficiently quenched the fluorescence due to electronic interaction. There was some steric distortion, too, because the size of this metal center was considerably smaller than the cavity of the porphyrin ring; thus, the macrocycle shrank a bit, resulting in its ruffled distortion.

### 2.2. Temperature-Affected Coordination and Spin-State Equilibrium

**Figure 5:**
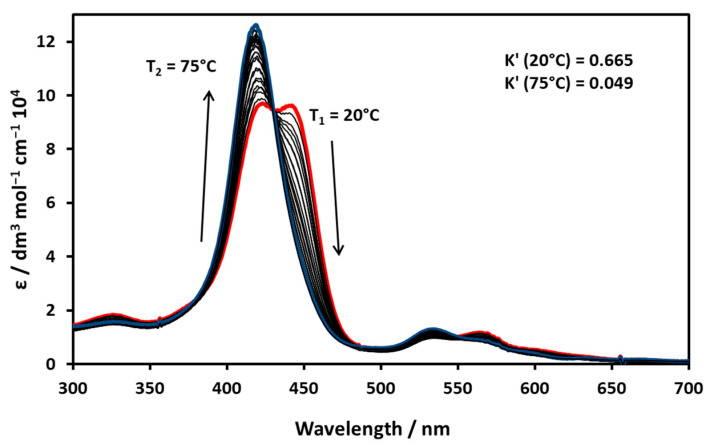
Change in absorption spectra of Ni(II)TMPyP^4+^ with increasing temperature in aqueous solution. The red spectrum belongs to 20 °C, the blue one to 75 °C.

The equilibrium of the spin state of Ni(II)TMPyP^4+^ in an aqueous solution was strongly affected by temperature variation, as shown in the absorption spectra (Figure 5). In the Soret range, a well-defined isosbestic point appeared at 430 nm, and with increasing temperature the equilibrium shifted towards the low-spin form. The nature of the isosbestic point suggests the axial coordination of a water molecule.

**Figure 6:**
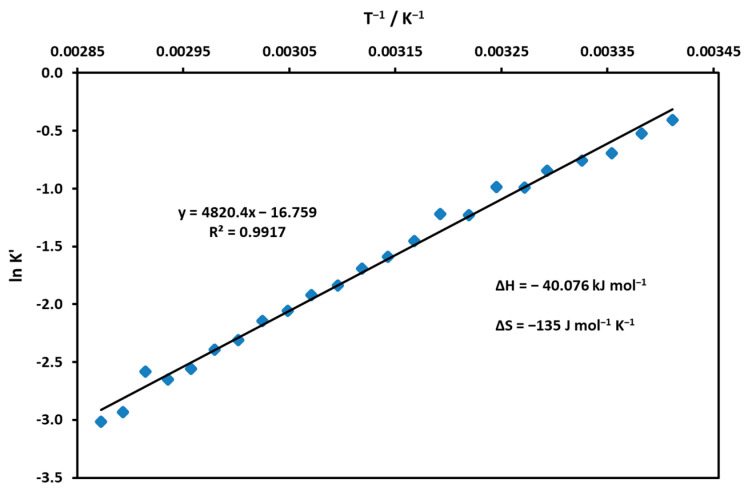
Thermodynamic data calculated for the coordination and spin-state equilibrium of Ni(II)TMPyP^4+^.

The composition of an equilibrium mixture was calculated for each temperature by using the molar absorptions of the low- and high-spin forms, and finally, the apparent equilibrium constants were calculated from the distribution of the partial mole fractions (K’ = C_high-spin_/C_low-spin_). By plotting the logarithm of the values for a given temperature as a function of 1/T, the enthalpy and entropy of the coordination reaction were determined (Figure 6). Based on the calculated thermodynamic constants, the process was associative (negative ΔS) and exothermic (moderately large negative ΔH).

### 2.3. Influence on the Coordination and Spin-State Equilibrium by Photoinduced Ligand Elimination

Excitation of this kind of Ni(II) porphyrin can result in the addition or elimination of an axial ligand to or from the coordination sphere of the metal center. An example of photo-induced ligand elimination was published by Ludwig et al. in the case of a five-coordinate complex [25]. In this case, however, the axial ligand was an azopyridine covalently attached to the porphyrin ring. Excitation caused a *cis-trans* isomerization at the azo unit, triggering de-coordination/coordination of the pyridine nitrogen. This observation suggests that a more weakly coordinated water molecule can be more easily eliminated from the axial position upon excitation of the Ni(II) complex, according to Equation (2), but photo-addition could not be excluded either (Equation (3)).

Ni(II)TMPyP^4+^(H_2_O) + hν → Ni(II)TMPyP^4+^ + H_2_O photo-elimination (2)

H_2_O + Ni(II)TMPyP^4+^ + hν → Ni(II)TMPyP^4+^(H_2_O) photo-addition (3)

For the photo-addition study, the equilibrium mixture of nickel porphyrins was illuminated at 405 nm, where the molar absorption coefficient of the low-spin complex was most different from that of the high-spin complex. No formation of the latter form was observed, i.e., no photo-addition of a water molecule took place.

**Figure 7:**
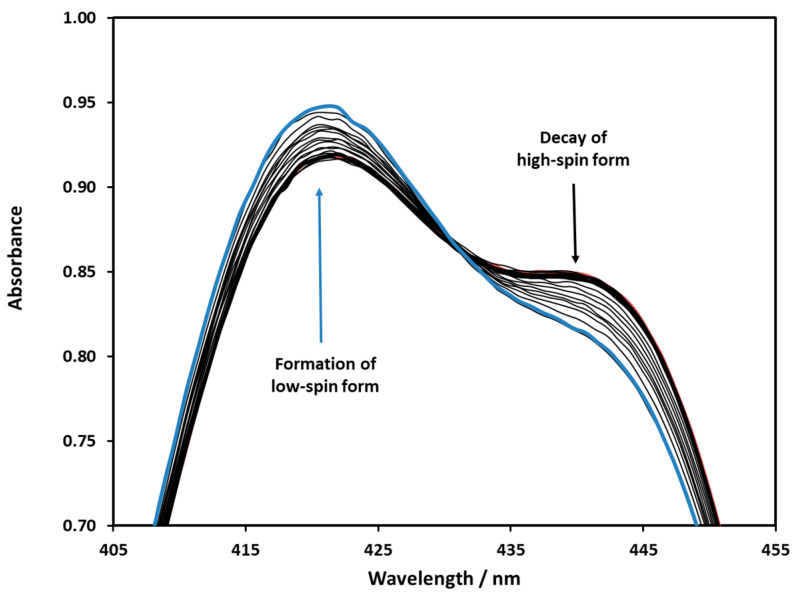
Spectral change in the Ni(II)TMPyP^4+^solution upon 455 nm irradiation. The blue spectrum was recorded at the end of irradiation.

To investigate photo-elimination, the equilibrium mixture of nickel porphyrins was illuminated at 455 nm, where the difference in molar absorption coefficients between the high- and low-spin complexes was the largest. The irradiation increased the amount of the latter, thus shifting the equilibrium towards the formation of the low-spin form (Figure 7).

**Figure 8:**
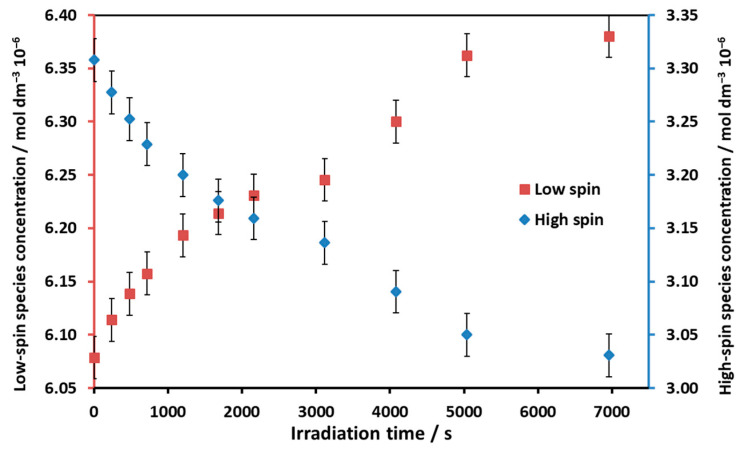
Concentration changes in the low- and high-spin forms during the 455 nm irradiation of the Ni(II)TMPyP^4+^ solution.

The concentrations of the low- and high-spin forms were calculated by using the individual molar absorbances of these species (Figure 8). According to the plots in Figure 8, the rate of the concentration changes of the high- and low-spin forms upon irradiation gradually decreased due to the thermal back reaction.

**Figure 9:**
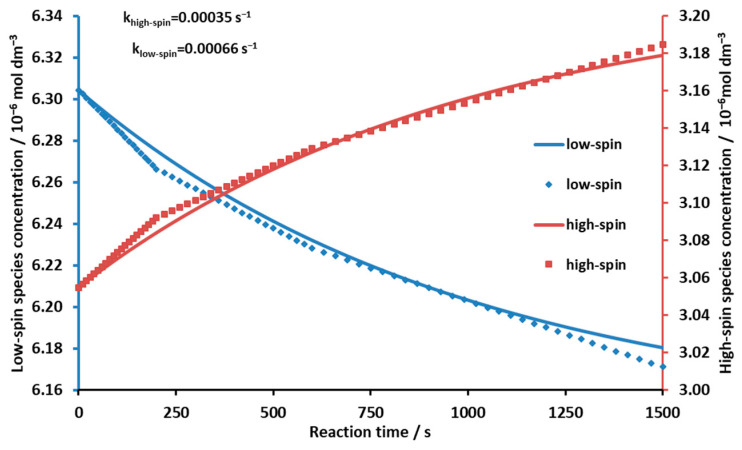
Kinetic curves of the back reaction after photo-elimination. The dots (markers) represent the measured concentrations, and the solid lines were calculated using the reaction rate constants.

After increasing the concentration (fraction) of the low-spin complex by illumination, the kinetics of the back reaction (“dark reaction”) could be studied. From the spectra taken during the measurement (using the molar of the individual species), their concentrations and the corresponding reaction rate constants could be determined (Figure 9). The reaction rate constant for the formation of the high-spin complex was 3.5 × 10^−4^ s^−1^, whereas that for the formation of the low-spin species was 6.6 × 10^−4^ s^−1^. (The latter was calculated by using the previous rate constant and the equilibrium constant, which is the ratio of the two rate constants). The measured points in Figure 9 did not fully fit our estimated kinetic curve, suggesting that axial ligand coordination and spin shift in the process may have been slightly separated in time. This was conspicuous in the initial 240 s period. The π-π* excitation can cause a weakening of the double-bond system conjugated in the ring, resulting in a slight expansion of the macrocycle. As a consequence, the high-spin central metal ion coordinated slightly above the plane of the porphyrin due to the axial ligand may still move closer to the mean plane of the macrocycle, which may trigger spin change before dissociation of the axial ligand. In other words, the photostationary state that was approached during the continuous irradiation may also have contained a certain proportion of complex forms in which spin change had already occurred but dissociation of the axial ligand had not yet (or not completely). The faster reversion of these complex forms may have been responsible for the higher rate in the initial period. The knowledge of the rate constant of the dark reaction (and the incident photon flux at the excitation wavelength) was necessary to calculate the quantum yield of the photo-elimination at the given wavelength of illumination, Φ_photo-elimination_ = 9.0 × 10^−5^.

### 2.4. Ground-State Interaction with TEOA

#### 2.4.1. Changes in the Absorption Spectrum of Ni(II)TMPyP^4+^ as a Function of TEOA

Since the previous photochemical studies indicated that this nickel(II) complex forms an associate with TEOA in ground-state equilibrium [12,24], spectral measurements were carried out to elucidate this interaction.

**Figure 10:**
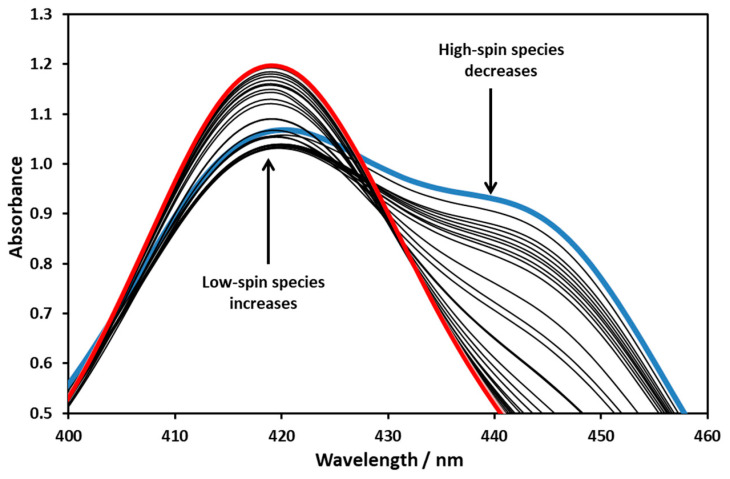
Spectral changes in the Soret band region for the Ni(II)TMPyP^4+^ solution with increasing TEOA concentration in the range of 0–0.5 M. The blue spectrum belongs to 0 M, the red one to 0.5 M.

As Figure 10 shows, the spectrum of the Ni(II)TMPyP^4+^ complex and its electronic structure changed upon the addition of TEOA. Weak interaction presumably resulted in the formation of an associate containing TEOA and water molecules. The equilibrium was reached within seconds. On the absorption spectra (Figure 10), it can be seen that, initially, the concentration of both the high- and the low-spin species decreased, and then the double peak disappeared and the peak corresponding to the low-spin complex became dominant.

**Figure 11:**
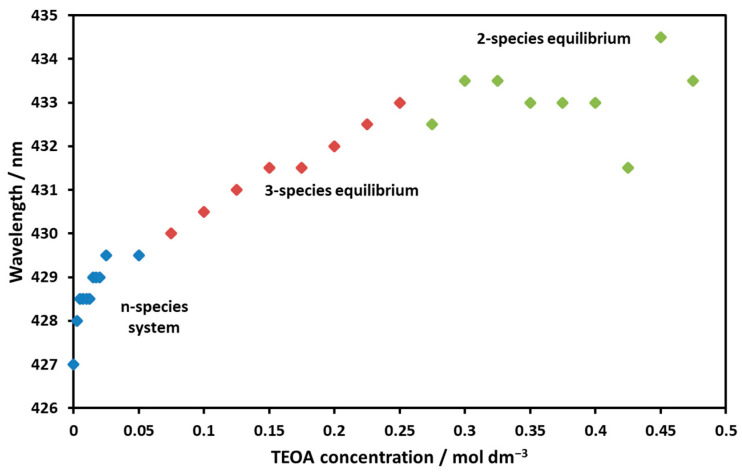
Migration of the isosbestic point’s wavelength in Figure 10 as a function of the TEOA concentration (C_Ni-porf_: 10^−5^ M, C_KCl_: 0.1 M).

It can be seen in Figure 10 that, deviating from the case of the temperature change, there was no sharp isosbestic point for the spectra. The addition of TEOA therefore resulted in the formation of more light-absorbing particles. After plotting the intersection of the spectra taken at the maximum (at 0.5 M) and different TEOA concentrations, a migration of and shift in the isosbestic point as a function of TEOA concentration was observed (Figure 11).

The obtained plot can be divided into three sections:

1. A steeply rising phase (0–0.05 M): Here, an n-particle system was assumed, which did not contain the final light-absorbing triethanolamine complex.
2. A linearly increasing phase (0.05–0.25 M): In this range, the isosbestic point migrated steadily, suggesting an equilibrium of three light-absorbing particles with increasing amounts of the final triethanolamine complex.
3. A constant range (0.25–0.5 M): The isosbestic point did not change, and two light-absorbing particles were present (as the value approached 0.5 M, the difference in the measured spectra became smaller and smaller, so their intersection became more uncertain).

#### 2.4.2. Characterization of the Ni(II)TMPyP^4+^–TEOA Associate

**Figure 12:**
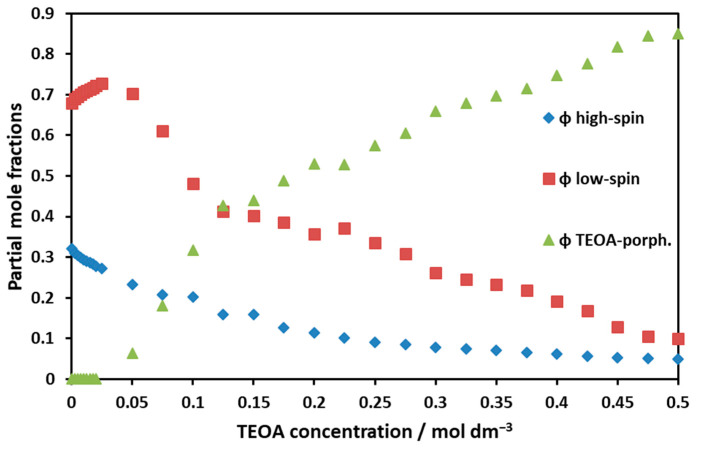
Partial mole fractions of different Ni(II)TMPyP^4+^ species as functions of the TEOA concentration.

The absorption spectrum of this associate was very similar to that of the low-spin square planar complex. The partial mole fractions of the low-spin and high-spin complexes as well as the TEOA associate as functions of the TEOA concentration (Figure 12) were calculated by using the corresponding molar absorbances (Figure S3). To estimate the equilibrium, the absorption spectra of triethanolamine porphyrin were estimated from the absorption spectra recorded at the highest TEOA concentration.

The total porphyrin concentration for a Ni(II)TMPyP^4+^–TEOA associate is provided by Equation (4).

C_Ni(II)TMPyP4+_ = [Ni(II)TMPyP^4+^] + [Ni(II)TMPyP^4+^(H_2_O)] + [Ni(II)TMPyP^4+^ (TEOA)] = [Ni(II)TMPyP^4+^] × (1 + K_H2O_ × [H_2_O] + K_TEOA_ × [TEOA]) (4)

Dividing Equation (4) by [Ni(II)TMPyP^4+^] yields the function 1/Φ_0_ = α(H_2_O; TEOA):

α(H_2_O; TEOA) = 1 + K_H2O_ × [H_2_O] + K_TEOA_ × [TEOA] (5)

**Figure 13:**
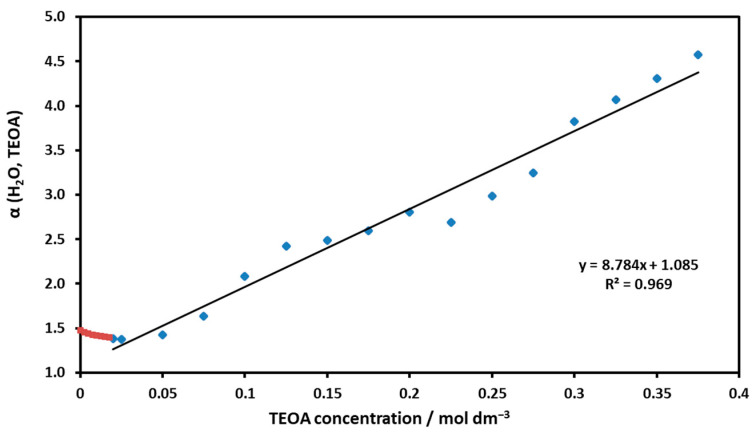
The slope of the line obtained as a function of the TEOA concentration per Equation (5) yields the equilibrium constant regarding the association of Ni(II)TMPyP^4+^ with TEOA.

Based on this relationship, the function α(H_2_O; TEOA) vs. [TEOA] was plotted in Figure 13.

Also, the equilibrium constants for TEOA binding, K_TEOA_ = 8.8 M^−1^, and water coordination K’(H_2_O) = 0.085 could be estimated. The latter suggests that the water coordination was reduced by TEOA (compared to K’(H_2_O) = 0.665; see Figure 6). The experimentally determined ΔG = −5.39 kJ mol^−1^ (at 25 °C) for the TEOA associate was smaller than the ~−10 kJ calculated for the axial coordination without solvent using quantum chemistry. The linear phase is preceded by a short descending series of dots (designated by red in Figure 13), indicating that the process was complex and did not proceed in a single step.

## 3. Materials and Methods

### 3.1. Materials

Nickel(II) sulfate, tetrastosylate salt 5,10,15,20-tetrakis(1-methylpyridinium-4-yl)porphyrin (H_2_TMPyP^4+^) as water-soluble porphyrin ligand, mercury(II) chloride, triethanolamine (TEOA), n-propanol, and cc. ammonia solution were purchased from Sigma-Aldrich (Budapest, Hungary). All of them were reagent-grade chemicals and were used without further purification. Tetrachloride salt of Ni(II)TMPyP^4+^ was purchased from Frontier Scientific (Newark, DE, USA). For actinometric measurements, 1,10-phenanthroline and potassium trisoxalatoferrate(III) were applied. The solvent used in this work was doubly distilled water treated with a Millipore Milli-Q system.

### 3.2. Preparation of Ni(II)TMPyP^4+^ Samples

Stock solutions of the cationic Ni(II)TMPyP^4+^ complex were prepared via in situ generation through the reaction of the corresponding free base and nickel(II) sulfate (in five-fold excess in a porphyrin concentration of 3 × 10^−5^ M) under air at room temperature. This reaction is extremely slow at r.t. [26]; thus, it was accelerated by the addition of HgCl_2_ at a very low concentration (10^−6^ M) and the temperature was increased to 70 °C. Even under these circumstances, the total conversion took about 10 days. The catalytic effect of Hg(II) in this reaction was based on its large ionic radius (102 pm [27]), which resulted in the formation of an out-of-plane intermediate Hg(II)-porphyrin [28]. Due to the distortion in this species, two diagonal pyrrolic nitrogen atoms become more accessible to another metal ion, even with a smaller ionic radius, on the other side of the macrocycle [29]. The behavior of the Ni(II)-porphyrin) obtained in this way was not affected by the presence of Hg(II) in the samples, which were prepared by 50–100 times dilution from the stock solution.

### 3.3. Methods

The UV-visible spectra were recorded on a Specord S-600 diode-array spectrophotometer (Analytik Jena, Jena, Germany). The emission measurements were carried out using Perkin-Elmer LS-50B (Waltham, MA, USA) and Fluoromax-4 (Horiba Jobin Yvon, Palaiseau, France) spectrofluorometers. (The latter was also equipped with a time-correlated single-photon counting unit for determination of fluorescence lifetimes). Illumination was performed with monochromatic light from a high-power (1000 W) mercury–xenon lamp (Oriel, Stratford, CT, USA) to measure the kinetics of photoelimination. Illuminations were realized with 3.5 cm^3^ solutions in 1 cm cells at r.t. and were continuously homogenized by magnetic stirring. Incident light intensity was determined by ferrioxalate actinometry [30]. Digital evaluations of the experimental data were carried out by various procedures using an MS Excel program. The applied mathematical relationships are included in the Supplementary Materials (as Text S1).

## 4. Conclusions

In our work, we confirmed that, in aqueous solution, the kinetically inert, in-plane complex (Ni(II)TMPyP^4+^), depending on the solvent and the presence of potential axial ligands, can exist in the forms of high-spin and low-spin complexes. In water, these species existing together in equilibrium display deviating photophysical properties. Soret band excitation of this metalloporphyrin in water shifted the equilibrium towards the low-spin species via the removal of an axially coordinated water molecule. The kinetics of the corresponding processes could also be measured. A temperature increase could also result in the dissociation of the water ligand. The binding of the electron donor TEOA led to the formation of a low-spin associate, reducing the coordination of water molecules. As observed earlier, this interaction promotes an efficient photoinduced electron transfer from TEOA to methyl viologen in aqueous systems containing this metalloporphyrin. Hence, these results can contribute to the optimization of such photocatalytic systems.

## Figures and Tables

**Table 1:** Parameters for the absorption bands of the low-spin Ni(II)TMPyP^4+^ complex (wavelengths of the band maxima, half-widths, and oscillator strengths). The designations of bands in the first line refer to the following: M and N are high-energy charge-transfer bands in the UV range, B(x,0) are Soret bands, and Q(y,0) are Q bands due to the transitions to the S_2_ and S_1_ excited electronic states with x and y vibronic states, respectively. The errors do not exceed 1%.

	M	N	B(1,0)	B(0,0)	Q(2,0)	Q(1,0)	Q(0,0)
**Wavelength/nm**	325	360	392	420	482	532	566
**w_1/2_/cm^−1^**	5280	1890	2090	1720	2360	952	1090
**Oscillator strength**	0.226	0.0518	0.195	0.825	0.0393	0.0412	0.0241

**Table 2:** Parameters for the absorption bands of the high-spin Ni(II)TMPyP^4+^ complex (wavelengths of the band maxima, half-widths, and oscillator strengths). The explanations for the band designations are provided in the caption of Table 1. The errors do not exceed 1%.

	M	N	B(1,0)	B(0,0)	Q(2,0)	Q(1,0)	Q(0,0)
**Wavelength/nm**	328	389	418	449	535	570	615
**w_1/2_/cm^−1^**	6220	2870	1490	1770	2210	1050	1180
**Oscillator strength**	0.515	0.244	0.198	0.938	0.0395	0.0419	0.0278

## Data Availability

The data presented in this study are available on request from the corresponding author. The data are not publicly available due to privacy.

## Supplementary Materials

The following supporting information can be downloaded at: https://www.mdpi.com/article/10.3390/molecules29020310/s1, Figure S1: Excitation and absorption spectra of the high-spin complex (in NH_3_ solution); Figure S2: Excitation and absorption spectra of the low-spin complex (in n-propanol system); Figure S3: Molar absorptivity spectra estimated for calculating the TEOA—Ni(II)-TMPyP^4+^ interaction; Text S1: Formulas used for evaluations.
