# Supplementary material for: Effects of Temperature, Axial Ligand, and Photoexcitation on the Structure and Spin-State of Nickel(II) Complexes with Water-Soluble 5,10,15,20-Tetrakis(1-methylpyridinium-4-yl)porphyrin
Source: Molecules. 2024 Jan 8;29(2):310. doi: 10.3390/molecules29020310 (PMC10818337; doi:10.3390/molecules29020310)
Supplement: Supplementary file 1 [file molecules-29-00310-s001.zip › molecules-2776581-supplementary.pdf]

Supplementary Materials

for

Effects of temperature, axial ligand, and photoexcitation on the structure and spin-state of nickel(II) complexes with water-soluble 5,10,15,20-tetrakis(1-methylpyridinium-4-yl)porphyrin

Máté Miklós Major, Zsolt Valicsek and Ottó Horváth *

Research Group of Environmental and Inorganic Photochemistry, Center for Natural Sciences, Faculty of Engineering, University of Pannonia, P.O.B. 1158, Veszprém H-8210, Hungary; miklos.mate.major@gmail.com (M.M.M.); valicsek.zsolt@mk.uni-pannon.hu (Z.V.).)

* Correspondence: horvath.otto@mk.uni-pannon.hu (O.H.); Tel+36-88-624-000 / 4585 ext.

Table of content

Figure S1..1

Figures S2, S3 2

Text S1..3

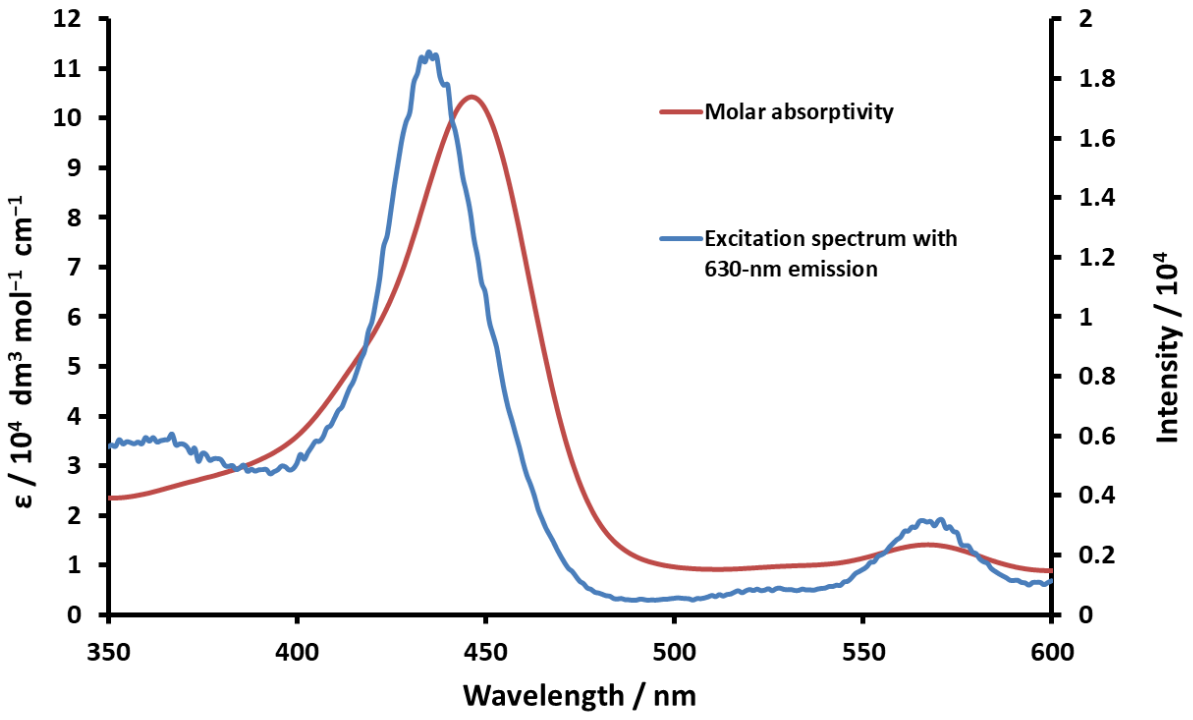

Figure S1. Excitation and absorption spectra of the high-spin complex (in NH_3 solution).

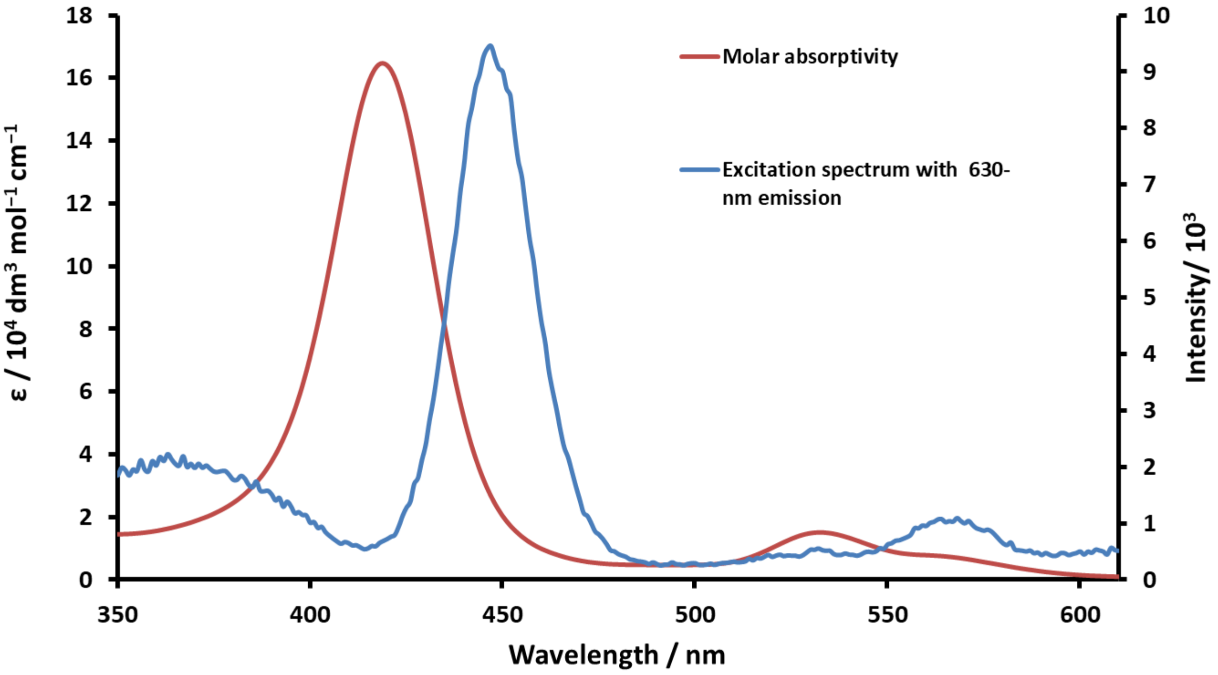

Figure S2. Excitation and absorption spectra of the low-spin complex (in n-propanol system).

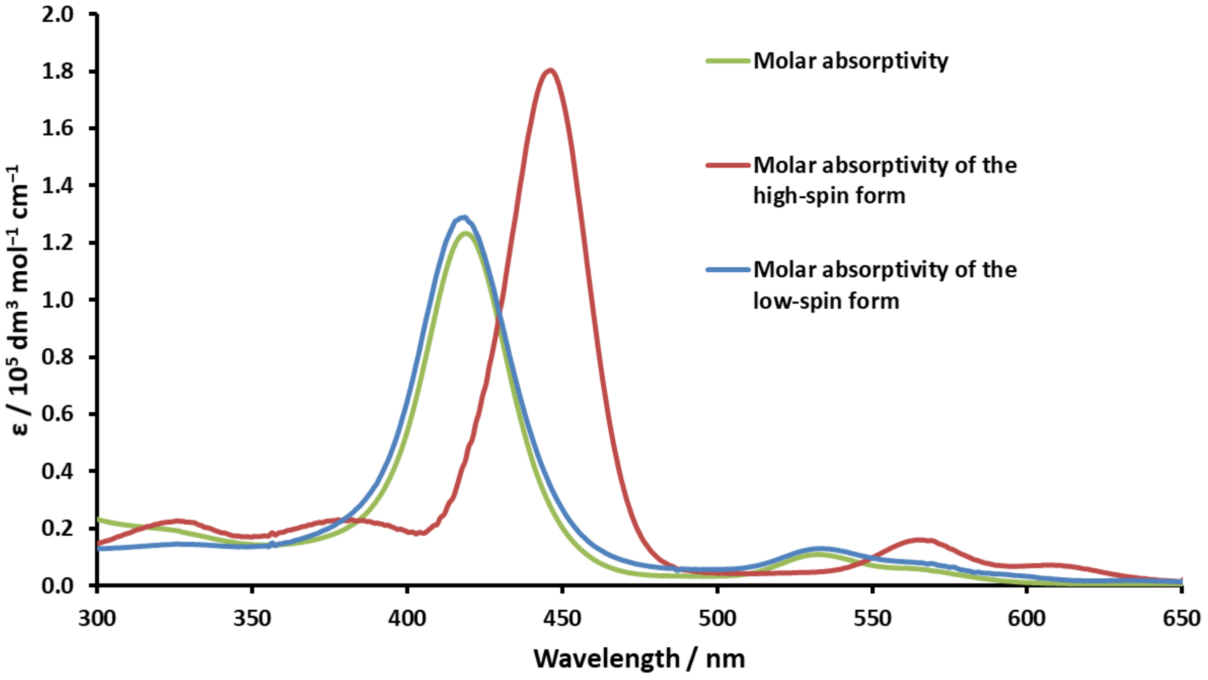

Figure S3. Molar absorptivity spectra estimated for calculating the TEOA—Ni(II)-$TMPyP^{4+}$ interaction.

Text S1

Formulas used for evaluations

1) Equation for the Gaussian curve (band):

$$A(\bar{\nu}) = \frac{A_{max}(\overline{\nu_0})}{exp\frac{(\bar{\nu} - \overline{\nu_0})^2}{2\sigma^2}} \tag{1}$$

where A(ν) is the absorbance (or intensity in the case of an emission spectrum) at the given wavenumber (ν) and its maximum (ν_0), respectively.

2) Oscillator strength:

$$f = (4.32 \times 10^{-9}\ \ Mcm^2)F \int \varepsilon(\omega)d\omega \tag{2}$$

where *f* is the oscillator strength, *F*=9n/$(n^2+2)^2$ is a factor dependent on the refractive index (n_{water} =1.33), *ε(ω)* is the molar absorptivity ($M^{-1}cm^{-1}$) at *ω* wavenumber.

3) Half-width (of a band):

$$w\,1/2 = \Delta\ = 2 \times 1.18\ \ \sigma \tag{3}$$

where σ is the standard deviation parameter of the Gaussian curve, and $\omega_{1/2}$ is its half-width.
